# Phylogenetics and Evolutionary Dynamics of Yunnan Acrididae Grasshoppers Inferred from 17 New Mitochondrial Genomes

**DOI:** 10.3390/insects16020151

**Published:** 2025-02-03

**Authors:** Keyao Zhang, Jing Song, Junhui Lu, Lu Zhao, Weian Deng, Delong Guan, Benyong Mao

**Affiliations:** 1 College of Life Sciences, Shaanxi Normal University, Xi’an 710119, China; keyaozh@163.com (K.Z.); 2023660007@hcnu.edu.cn (J.S.); lujunhui@snnu.edu.cn (J.L.); zl609677869@snnu.edu.cn (L.Z.); 2 Guangxi Key Laboratory of Sericulture Ecology and Applied Intelligent Technology, Hechi University, Hechi 546300, China; dengweian5899@163.com; 3 Guangxi Collaborative Innovation Center of Modern Sericulture and Silk, Hechi University, Hechi 546300, China; 4 College of Agriculture and Biological Science, Dali University, Dali 671003, China

**Keywords:** mitochondrial genome, molecular evolution, phylogenetic relationships, adaptive radiation, Acrididae, Yunnan biodiversity, selective pressure, taxonomic revision, species diversification

## Abstract

Yunnan Province, a global biodiversity hotspot, hosts a diverse array of Acrididae grasshoppers essential for ecosystem dynamics and agriculture. To elucidate the phylogenetic relationships and evolutionary history of this group, we sequenced and analyzed complete mitochondrial genomes from 17 Acrididae species endemic to Yunnan, with genome lengths ranging from 15,403 to 15,943 base pairs. These data were integrated with mitochondrial sequences from 46 additional species to construct comprehensive phylogenetic trees. The maximum-likelihood tree identified four major clades with robust support (bootstrap values > 90%), revealing significant lineage diversification during the Early Eocene (51.94 million years ago, Mya) and subsequent radiations in the Miocene (~20 Mya) and Pliocene (~5 Mya). These divergence times correlate with major geological events and climatic shifts in the region, such as the uplift of the Tibetan Plateau and the intensification of the Asian monsoon. Notably, several species within the Coptacrinae and Oxyinae subfamilies, particularly *Pseudodotraulia cornuata* and *Spathosternum prasiniferum*, were found to be polyphyletic, indicating the necessity for taxonomic revisions. Further cluster analyses of codon usage bias and genetic distance support these taxonomic revisions within Acrididae. This robust phylogenetic framework underpins conservation strategies aimed at preserving Yunnan’s rich grasshopper biodiversity and informs updates to their phylogeny.

## Simple Summary

Yunnan Province in southwestern China is home to a rich variety of grasshoppers, particularly from the Acrididae family, which play key roles in ecosystems and agriculture. However, understanding the phylogenetic relationships and evolution of these grasshoppers has been difficult using just their physical traits. To gain deeper insights, our study analyzed the mitochondrial DNA—the genetic material passed from mothers to offspring—of 17 different Acrididae species found in Yunnan. By comparing these new genetic sequences with those from 46 other Acrididae species, we were able to construct a detailed family tree showing how these grasshoppers are related. Our findings revealed that some groups previously thought to be closely related are not, suggesting that the existing classification needs to be updated. Additionally, we determined that the diversification of these grasshoppers occurred alongside significant geological and climate changes in the region over 50 million years ago. This research not only clarifies the evolutionary history of Yunnan’s grasshoppers but also provides essential information for conserving their diverse populations. By improving our understanding of these insects, we can probe into their accurate phylogeny.

## 1. Introduction

The Acrididae family, commonly known as short-horned grasshoppers, represents one of the most diverse and ecologically significant groups within the Orthoptera order [1,2]. With over 10,000 described species, Acrididae exhibits remarkable morphological, behavioral, and ecological diversity, allowing these insects to inhabit a wide range of environments, from arid deserts to lush montane forests [3,4]. This diversity not only underscores the evolutionary success of Acrididae but also highlights its pivotal role in various ecosystems as primary consumers, pest species, and indicators of environmental health.

Systematic biology, which encompasses the study of the diversification of life and the relationships among living organisms through time, is fundamental to understanding the evolutionary processes that generate and maintain biodiversity [5]. Within this framework, the integration of molecular data, particularly mitochondrial genomes, has revolutionized the resolution of phylogenetic relationships, offering unprecedented insights into the evolutionary history and taxonomy of diverse taxa. Mitochondrial DNA (mtDNA) is especially valuable in systematics due to its typically rapid rate of evolution, maternal inheritance, and lack of recombination, which facilitate the detection of genetic differences among closely related species [4,6]. The complete mitochondrial genome provides a comprehensive set of genetic markers, including protein-coding genes, ribosomal RNA genes, and transfer RNA genes, which collectively offer a robust framework for reconstructing evolutionary relationships. Comparative mitochondrial genomics allows for the identification of conserved and variable regions, enabling the assessment of both deep and shallow phylogenetic divergences [7,8].

Yunnan Province, located in southwestern China, is recognized as one of the world’s most biodiverse regions [9,10]. Its complex topography, characterized by towering mountain ranges, deep river valleys, and diverse climatic zones, creates a mosaic of habitats that support a high degree of species richness and endemism [11,12,13]. The province lies at the crossroads of several major biogeographic regions, including the Palearctic, Oriental, and Indo-Malayan zones, facilitating the convergence of diverse faunal elements [14,15]. The unique geological history of Yunnan, marked by the uplift of the Tibetan Plateau and subsequent climatic fluctuations, has played a crucial role in shaping its rich biodiversity [12,16]. These geological events have induced significant habitat heterogeneity and environmental gradients, promoting both allopatric and ecological speciation [15,17]. Consequently, Yunnan serves as an ideal natural laboratory for studying the mechanisms of diversification and the evolutionary processes that generate and sustain high levels of biodiversity.

Despite the ecological and evolutionary significance of Acrididae, the systematic understanding of this family remains incomplete and, in many cases, contentious [18,19]. Traditional taxonomy, which relies heavily on morphological traits, has laid the groundwork for understanding these insects. However, the field of Acrididae taxonomy itself is facing significant challenges. The expertise needed for precise taxonomic work demands extensive training and a long period of apprenticeship, making it a highly specialized field. Over time, as older generations of taxonomists retire, there has been a noticeable decline in individuals taking up this discipline [20]. The transition among generations has notably impacted the study of the Acrididae family, with all authors of this paper having firsthand experience of these changes. The high species diversity combined with the morphological similarities among different taxa makes it challenging to establish clear taxonomic boundaries, pushing the shift towards the use of molecular markers as an essential tool. Initial molecular research has started to clarify the complex phylogenetic relationships within Acrididae, uncovering cases of polyphyly and paraphyly in what were once considered well-defined subfamilies and genera [21,22,23]. These discoveries highlight the critical need for thorough molecular investigations to enhance and refine the traditional morphological classifications. Yet, the limitations of current research are evident, with issues like inadequate taxon sampling, incomplete mitochondrial genome data, and the dependence on a limited number of genetic markers, all of which hinder the ability to fully resolve the intricate evolutionary relationships within this family [4,6,19].

In response to the outlined challenges and opportunities, this study seeks to enhance the systematic and evolutionary comprehension of the Acrididae family in Yunnan Province through an extensive analysis of mitochondrial genomes. A primary objective is the sequencing and assembly of complete mitochondrial genomes for 17 Acrididae species native to Yunnan, ensuring a broad representation across various subfamilies and genera within the family. This comprehensive mitochondrial genomic dataset will serve as a solid genetic foundation for subsequent phylogenetic and comparative analyses. Building upon these foundational objectives, the study also aims to propose necessary taxonomic revisions based on the integrated phylogenetic and genetic findings, ensuring that current classification systems accurately reflect evolutionary relationships. By refining the taxonomic framework, the research will facilitate more precise biodiversity assessments and enhance the effectiveness of conservation strategies. The implications of these findings extend to biodiversity conservation in Yunnan, where the study will emphasize the importance of accurate taxonomic classifications and evolutionary insights in informing and guiding conservation efforts. Furthermore, by providing a detailed understanding of Acrididae diversity and evolution, the research aims to support the preservation of genetic diversity and ecological functions of these grasshoppers in Yunnan’s unique and biodiverse ecosystems.

Moreover, this research can inform biodiversity conservation strategies by revealing patterns of co-evolution and ecological associations among different grasshopper lineages [24]. Species that share recent common ancestors often have similar ecological requirements, and protecting representative habitats for phylogenetically diverse groups can help maintain both species diversity and ecosystem functions. This is particularly relevant in Yunnan, where many Acrididae species serve as indicators of grassland health and play crucial roles in nutrient cycling. By refining the taxonomic framework through molecular phylogenetics, this research will facilitate more precise biodiversity assessments and enhance the effectiveness of conservation strategies [25]. The study aims to identify evolutionarily distinct lineages that may require priority conservation attention and provide insights into how historical processes have shaped current biodiversity patterns. This information will be crucial for developing targeted conservation measures that consider both the evolutionary history and current ecological requirements of Yunnan’s grasshopper fauna.

## 2. Materials and Methods

### 2.1. Sample Collection and DNA Extraction

Specimens representing 17 Acrididae species were collected from various locations across Yunnan Province, China, between 2018 and 2023 by Prof. Maobenyong’s team at Dali University (Table 1). Collection sites covered diverse ecological zones across the entire Yunnan Province which do not have major biogeographic zones. However, those species showed strong zone specificity—for example, *Eyprepocnemis yunnanensis* was found exclusively in the southern region of Yunnan, while *Ranacris jinpingensis* was restricted to Jinping County. Collection sites for these species were strategically selected to represent all three major biogeographic zones within Yunnan: The northwestern localities of Dali and Baoshan, the southeastern localities of Jinping and Mengla, and the southern valleys of Jinghong and Ruili. For each species, over three individuals were collected. All specimens were morphologically identified using established taxonomic keys and preserved in 100% ethanol at −80 °C. For DNA sequencing preparation, one sample with the largest body size was selected from each species collection. Total genomic DNA was extracted from the muscle tissue of the femur using the TIANamp Genomic DNA Kit (TIANGEN, Beijing, China. DP304-03) following the manufacturer’s protocols.

### 2.2. DNA Quality Control and Genome Sequencing

The quality and quantity of extracted DNA were assessed using a NanoDrop 2000 spectrophotometer (Thermo Scientific, Waltham, MA, USA) and Qubit 2.0 fluorometer (Life Technologies, Carlsbad, CA, USA). DNA integrity was evaluated by 1% agarose gel electrophoresis. Library preparation was performed using the TruSeq DNA PCR-Free Library Preparation Kit (Illumina, FC-121-3003, San Diego, CA, USA) following the manufacturer’s instructions. Briefly, genomic DNA was fragmented to a target size of 350 bp using a Covaris S220 focused ultrasonicator. The resulting fragments were end-repaired, A-tailed, and ligated with Illumina adapters. Paired-end sequencing (2 × 150 bp) was performed on an Illumina HiSeq 2500 platform at Novogene Co., Ltd. (Beijing, China). Raw reads were quality-filtered using Trimmomatic v0.39 with the following parameters: LEADING:3 TRAILING:3 SLIDINGWINDOW:4:15 MINLEN:36.

### 2.3. Mitochondrial Genome Assembly and Annotation

Clean reads were used for mitochondrial genome assembly using NOVOPlasty v4.2.1 [26] with default parameters. The complete mitochondrial genome sequence from a closely related species, *Oxya chinensis* (Genbank ID: EF437157), was used as the seed sequence for initial assembly. The circular nature of the mitochondrial genomes was verified by examining the assembly graphs. The assembled mitochondrial genomes were annotated using the MITOS2 web server [27] with the invertebrate mitochondrial genetic code. Gene boundaries were manually curated based on start and stop codons to minimize intergenic spaces and gene overlaps in Geneious Prime (v2024).

### 2.4. Comparative Genomic and Phylogenetic Analyses

Complete mitochondrial genomes were aligned using MAFFT v7.487 [28] with the G-INS-i strategy. Nucleotide composition and codon usage were analyzed using Phylosuite v1.2.2 [29]. For the phylogenetic analysis, we integrated our newly obtained mitochondrial genome sequences with 46 previously published sequences of Acrididae from the Yunnan region, which were retrieved from GenBank. Three species from the Tetrigidae family were chosen as outgroups. The GenBank accession numbers for these sequences are listed in Table S1. Individual genes were aligned separately and concatenated also using Phylosuite v1.2.2 [29]. Model testing was performed using ModelFinder implemented in IQ-TREE v2.1.3 [30]. Maximum likelihood (ML) phylogenetic analysis was conducted using IQ-TREE with 1000 ultrafast bootstrap replicates. Divergence times were estimated using BEAST v2.6.6 [31] with a relaxed, uncorrelated lognormal clock model. A Yule process was used as the tree prior. Two independent MCMC runs were performed for 10 million generations, sampling every 10,000 generations. Convergence was assessed using Tracer v1.7.2 [32]. TreeAnnotator v2.6.6 [33] was used to generate the maximum clade credibility tree after discarding 10% burn-in. The tree was calibrated using fossil evidence and previously published divergence times, with key calibration points, including the split between Tetrigoidea and Acridomorpha (114.971 Mya, 95% HPD = 103.662–123.045 Mya) [3] and the divergence of Acridoidea and Pyrgomorphoidea (71.468 Mya, 95% HPD = 59.981–82.081 Mya) [2].

### 2.5. Statistical Analysis and Visualization

Codon usage bias was analyzed using MEGA v12 [34] to calculate the relative synonymous codon usage (RSCU) values. Pairwise genetic distances among species were calculated also using MEGA v12 [34] under the Kimura 2-parameter model with gamma-distributed rate variation. Principal component analysis (PCA) of genetic distances was performed using the ‘ade4’ package in R [35]. The Statistical analyses were performed using R v4.1.0. The Mann–Whitney U test was used to compare genomic features among groups. Correlation analyses were conducted using Spearman’s rank correlation coefficient. The significance of clustering patterns was tested using ANOSIM implemented in the ‘vegan’ package [36]. Multiple testing corrections were performed using the Benjamini-Hochberg method. Graphics were generated using the online OmicStudio platform (www.omicstudio.cn, accessed on 23 November 2024).

## 3. Results

### 3.1. Mitochondrial Genome Organization and Features

**Figure 1:**
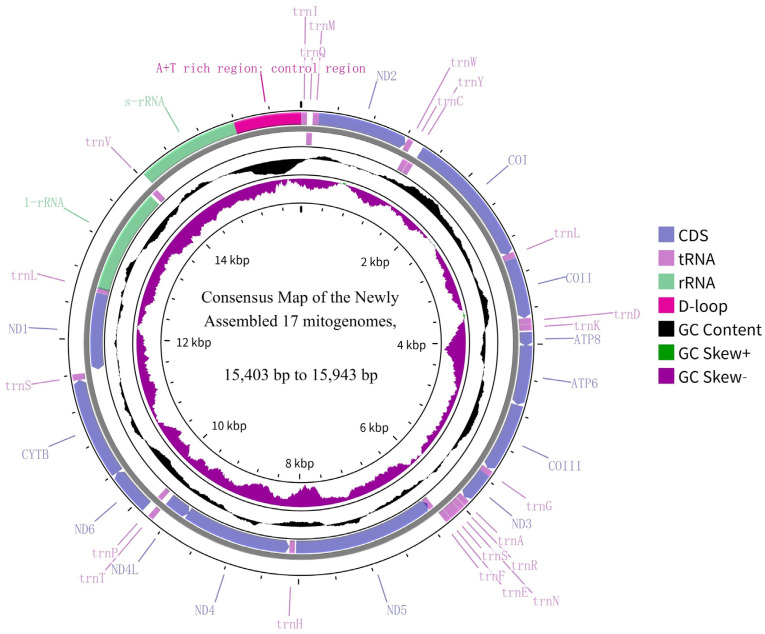
Consensus map of mitochondrial genome organization in 17 Yunnan Acrididae species. The circular genome (15,403–15,943 bp) contains 37 genes, including protein-coding genes (PCGs, blue), transfer RNA genes (tRNAs, pink), and ribosomal RNA genes (rRNAs, green). The D-loop region is shown in magenta. The inner circles display GC content (black) and GC skew (purple/green). Gene transcription direction is indicated by position relative to the circle (outside: forward strand; inside: reverse strand). The A + T-rich control region and other notable features are labeled. All 17 species share this highly conserved gene arrangement, typical of insect mitochondrial genomes.

We successfully assembled and annotated complete mitochondrial genomes for all 17 Acrididae species, with sizes ranging from 15,403 bp (*Pseudoptygonotus prominemarginis*) to 15,943 bp (*Heteropternis rufipes*) (Figure 1). All mitogenomes exhibited the canonical metazoan mitochondrial structure, containing 37 genes: 13 protein-coding genes (PCGs), 22 transfer RNA genes (tRNAs), and two ribosomal RNA genes (rRNAs). Notably, the gene arrangement was strictly conserved across all studied species, maintaining the ancestral insect mitochondrial genome organization without any evidence of tRNA duplications or rearrangements (Figure 1). This high degree of synteny suggests strong evolutionary constraints on mitochondrial genome organization in Acrididae despite their significant morphological and ecological diversification in different areas, including Yunnan Province.

**Figure 2:**
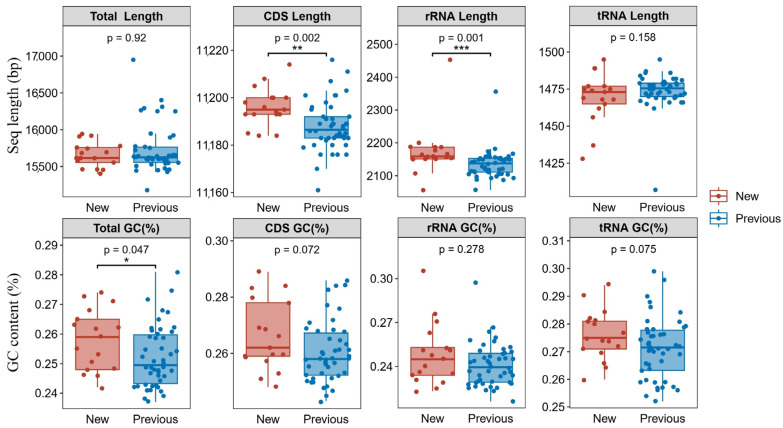
Comparison of mitochondrial genome features between newly sequenced (*n* = 17) and previously reported (*n* = 46) Acrididae species from Yunnan Province. Box plots show the distribution of sequence lengths (bp) and GC content (%) for total mitogenomes and specific genomic regions (CDS, rRNA, and tRNA). Red boxes represent newly sequenced species, while blue boxes indicate previously reported species. Box boundaries show the interquartile range, with the middle line indicating the median. Whiskers extend to the most extreme data points within 1.5 times the interquartile range. Individual data points are overlaid to show the distribution of values. Statistical significance levels are indicated as *** *p* < 0.001, ** *p* < 0.01, and * *p* < 0.05. *p*-values were calculated using two-tailed Mann–Whitney U tests.

Comparative analysis among our 17 newly sequenced mitogenomes and 46 previously reported Acrididae species from Yunnan revealed several notable features (Figure 2). While the total mitogenome lengths showed no significant difference between new and previous sequences (*p* = 0.92), we observed significant variations in specific genomic regions. The protein-coding sequences (CDS) in our newly sequenced species were significantly longer (*p* = 0.002) than those previously reported, with an average difference of approximately 20 bp. Similarly, the ribosomal RNA genes showed marked length variation (*p* = 0.001), with our new sequences generally containing longer rRNA regions. Interestingly, the tRNA genes maintained consistent lengths across all species (*p* = 0.158), suggesting strong structural constraints on these essential RNA molecules.

The nucleotide composition analysis revealed subtle but significant differences in GC content between new and previously reported sequences. The total GC content in our newly sequenced mitogenomes was slightly higher (*p* = 0.047), although this pattern varied across different genomic regions. The CDS and tRNA regions showed marginally higher GC content in new sequences (*p* = 0.072 and *p* = 0.075, respectively), while the rRNA regions maintained similar GC levels (*p* = 0.278) between both groups. These compositional variations might reflect adaptation to different environmental conditions or evolutionary constraints across Yunnan’s diverse ecological niches.

### 3.2. Phylogenetic Inference and Divergence Time Estimation

**Figure 3:**
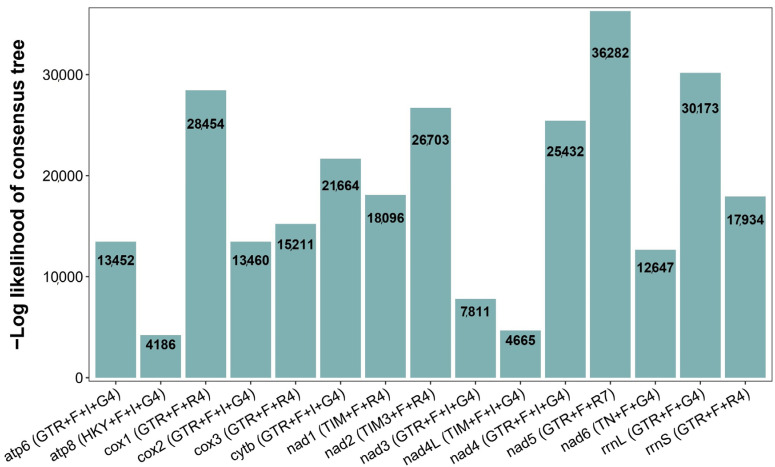
Best-fit evolutionary models and phylogenetic signal strength for mitochondrial genes in Yunnan Acrididae species. Bar heights represent the negative log-likelihood values obtained from IQ-TREE analyses. The best-fit substitution model for each gene is shown in parentheses.

As mitochondrial genomes serve as crucial molecular markers for phylogenetic inference, we aimed to reconstruct the phylogenetic relationships among Acrididae species in Yunnan. We first evaluated the phylogenetic signal and evolutionary models for each mitochondrial gene. Model testing using IQ-TREE revealed that the GTR + F + R4 was the most frequently selected as the best-fit model, followed by GTR + F + G4 (Figure 3). The negative log-likelihood values varied substantially among genes, ranging from 4186 (*atp8*) to 36,282 (*nad5*), indicating considerable differences in phylogenetic information content among different mitochondrial genes. Notably, the *nad* gene family, particularly *nad5*, *nad4*, and *nad1*, showed the highest-log likelihood values, suggesting these genes contain stronger phylogenetic signals for resolving relationships within Acrididae.

**Figure 4:**
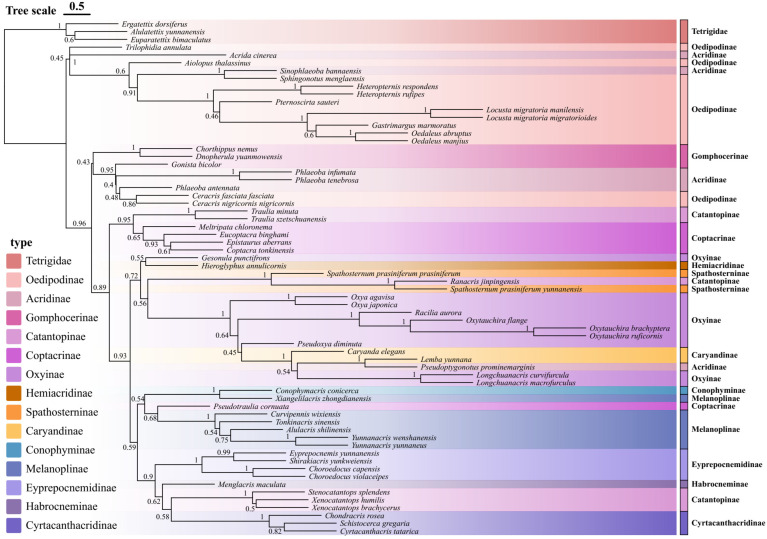
Species tree of Yunnan Acrididae based on concatenated mitochondrial genes. The tree was constructed using ASTRAL from individual gene trees generated by IQ-TREE maximum likelihood analyses. Numbers at nodes represent posterior probability values.

Based on the individual gene trees generated by IQ-TREE analyses, we employed ASTRAL-III to reconstruct a species tree that accounts for potential gene tree discordance. The resulting species tree revealed several well-supported monophyletic clades and provided new insights into the evolutionary relationships among Yunnan Acrididae species (Figure 4). The tree topology reveals the major clades with high support values (>0.9). In detail, members of the subfamily Oedipodinae, including *Pternoscirta sauteri* and *Heteropternis rufipes*, form a monophyletic group. This clade is sister to a diverse assemblage containing representatives from Catantopinae and Eyprepocnemidinae, including *Xenocatantops humilis* and *Eyprepocnemis yunnanensis*, with strong nodal support (0.95).

A surprising finding emerged regarding the placement of *Conophymacris conicerca*, which forms a distinct lineage separate from its traditionally assigned subfamily members of Conophyminae. This suggests a possible need for taxonomic revision of this group. The Coptacrinae representatives, including *Eucoptacra binghami*, *Epistaurus aberrans*, and *Coptacra tonkinensis*, cluster together with high support (0.97), confirming their close evolutionary relationships and current taxonomic placement. Our analysis also provides strong evidence for the monophyly of the *Spathosternum*-*Racilia* clade (support value 0.93), though their relationship to other groups differs from traditional morphology-based classifications. The newly sequenced *Longchuanacris macrofurculus* and *Lemba yunnana* form a well-supported clade (0.96), suggesting a closer evolutionary relationship than previously recognized. These findings demonstrate the utility of mitochondrial genomic data in resolving phylogenetic relationships and highlight the need for continued revision of grasshopper systematics, particularly within the diverse fauna of Yunnan Province.

**Figure 5:**
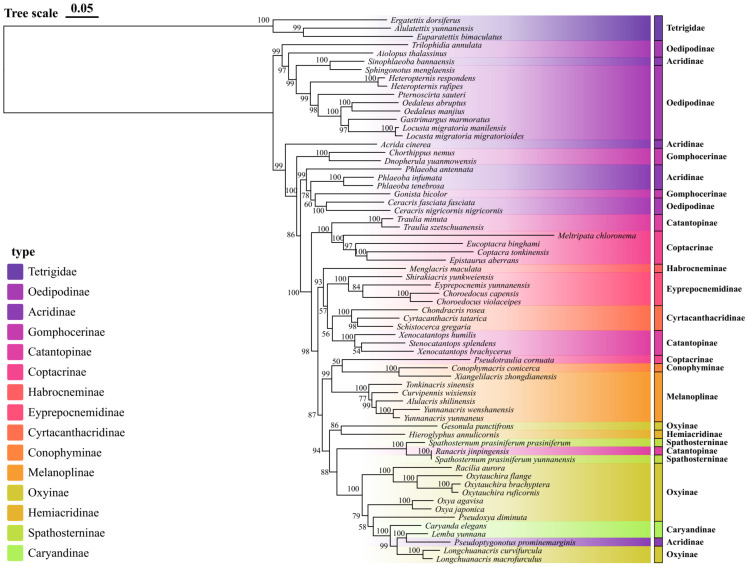
Maximum likelihood phylogenetic tree of Yunnan Acrididae based on concatenated mitochondrial sequences. Branch support values are shown as ultrafast bootstrap percentages (UFboot). Major subfamilies are indicated by different colors.

Although the ASTRAL analysis offered valuable insights into species relationships, it showed moderate support for several critical nodes, likely due to factors such as incomplete lineage sorting and varied evolutionary rates among different mitochondrial genes. To overcome this issue, we conducted an additional analysis using the maximum likelihood method on the concatenated sequences of all mitochondrial genes, which provided well-supported monophyletic clades with higher bootstrap values (see Figure 5). The analysis, incorporating both newly sequenced mitochondrial genomes and previously published data, revealed several noteworthy evolutionary patterns and relationships among grasshopper species from Yunnan Province.

The phylogenetic reconstruction demonstrates that it has uplifted most of the hierarchical clades with strong bootstrap support (>90%). The Oedipodinae clade, including *Locusta*, *Gastrimargus*, *Oedaleus*, and related genera, is strongly supported (97–100%). Within this group, the two subspecies of *Locusta migratoria* (*L. m. manilensis* and *L. m. migratorioides*) show an extremely close relationship with very short branch lengths. Another well-supported clade (100%) comprises the *Oxya* species (*O. agavisa* and *O. japonica*), sister to *Pseudoxya diminuta*. The *Oxytauchira* species form a monophyletic group with 100% bootstrap support, with *O. brachyptera* and *O. ruficornis* being more closely related to each other than to *O. flange*. Notably, *Spathosternum prasiniferum* subspecies cluster together with *Ranacris jinpingensis* with high support (100%), suggesting they belong to a clade that requires further merging. The genus *Yunnanacris* (*Y. wenshanensis* and *Y. yunnaneus*) also forms a well-supported clade (100%), sister to *Alulacris shilinensis* (99%).

A particularly interesting finding emerges from the central portion of the tree, where we observe an unexpected sister-group relationship between *Spathosternum prasiniferum* and members of the Oxyinae. This relationship, supported by high bootstrap values (93%), suggests a need to reconsider the current taxonomic placement of these taxa. The analysis also reveals a well-supported clade (bootstrap value 98%) containing *Xenocatantops humilis* and related species, confirming their close evolutionary relationships while highlighting several novel internal arrangements. The most derived clade in our analysis shows strong support for the grouping of *Longchuanacris macrofurculus* with other recently described Yunnan endemics. This placement provides the first robust phylogenetic framework for understanding the evolution of these regional specialists. Notable within this group is the position of *Lemba yunnana*, which forms a distinct lineage with implications for understanding local speciation patterns and biogeographic history. Moreover, our results also highlight several instances of phylogenetic incongruence with traditional classification schemes. For example, members of the Coptacrinae (including *Eucoptacra binghami* and *Coptacra tonkinensis*) appear in different positions across the tree, suggesting that this subfamily may not be monophyletic as currently defined. Similarly, the positions of several Catantopinae representatives indicate that this large subfamily may require taxonomic revision.

**Figure 6:**
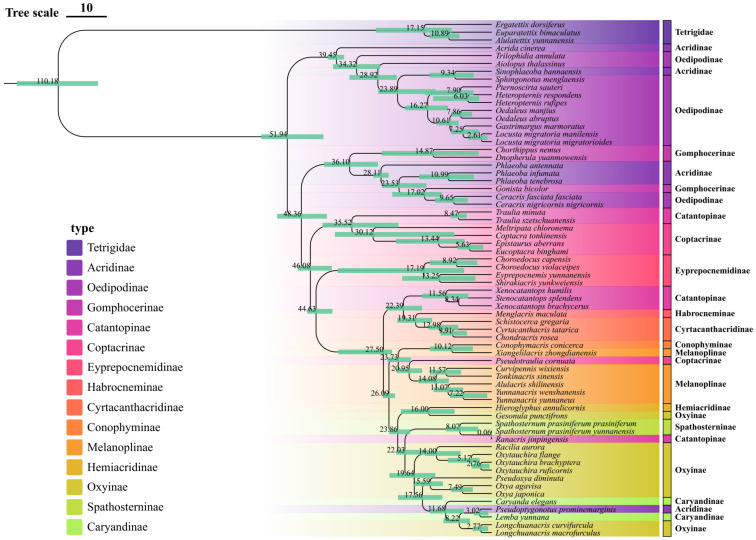
Time-calibrated phylogeny of Yunnan Acrididae based on concatenated mitochondrial sequences (13 PCGs + 2 rRNAs) using BEAST2. Node ages are shown in millions of years ago (Mya), with blue bars indicating 95% highest posterior density (HPD) intervals.

Molecular dating analysis using BEAST2 revealed the temporal framework of grasshopper evolution in Yunnan Province, with most major diversification events occurring during the Cenozoic era (Figure 6). The crown time for the Yunnan Acrididae is estimated to be around 51.94 million years ago (Mya). The time-calibrated phylogeny provides crucial insights into the timing and pattern of grasshopper radiation in this biodiversity hotspot. In detail, our analysis indicates that the initial divergence among the major lineages occurred approximately 51–45 Mya during the Early Eocene, coinciding with a period of global warming and significant geological activity in the region. This basal split gave rise to two major clades, with the first radiation including members of the Oedipodinae and related groups. The second major radiation, occurring around 40–35 Mya during the Late Eocene, led to the diversification of several contemporary lineages, including the Catantopinae and Eyprepocnemidinae.

The middle Oligocene (approximately 28–23 Mya) marked another significant period of diversification, particularly evident in the radiation of the *Spathosternum*-*Racilia* clade. This timing correlates with major geological events in the Yunnan region, including the uplift of the Himalayan-Tibetan Plateau and the formation of the modern Asian monsoon system. The divergence of *Xenocatantops humilis* and its allies occurred during this period, suggesting that these geological and climatic changes may have played a crucial role in driving speciation events.

More recent diversification events are observed in the terminal branches, with multiple speciation events occurring during the Miocene (23–5.3 Mya) (Figure 6). Notably, the split among *Longchuanacris macrofurculus* and related taxa occurred approximately 15–10 Mya, corresponding to a period of intense monsoon activity and habitat diversification in Yunnan. The youngest divergence events, dating to the Pliocene–Pleistocene transition (around 5.63 Mya), are particularly evident in the Coptacrinae clade, suggesting recent speciation possibly driven by quaternary climatic oscillations. The temporal pattern of diversification reveals several periods of increased speciation rates, particularly during the Oligocene–Miocene transition (23 Mya) and the Mid-Miocene Climatic Optimum (15 Mya). These periods coincide with major environmental and geological changes in Southeast Asia, suggesting that the evolution of Yunnan grasshoppers was strongly influenced by historical climate change and tectonic events. The dating analysis also reveals that many endemic species originated during periods of significant environmental change, highlighting the importance of historical processes in shaping current biodiversity patterns in the region.

### 3.3. Phylogenetic Implications Through Analysis of Codon Usage and Genetic Distances

**Figure 7:**
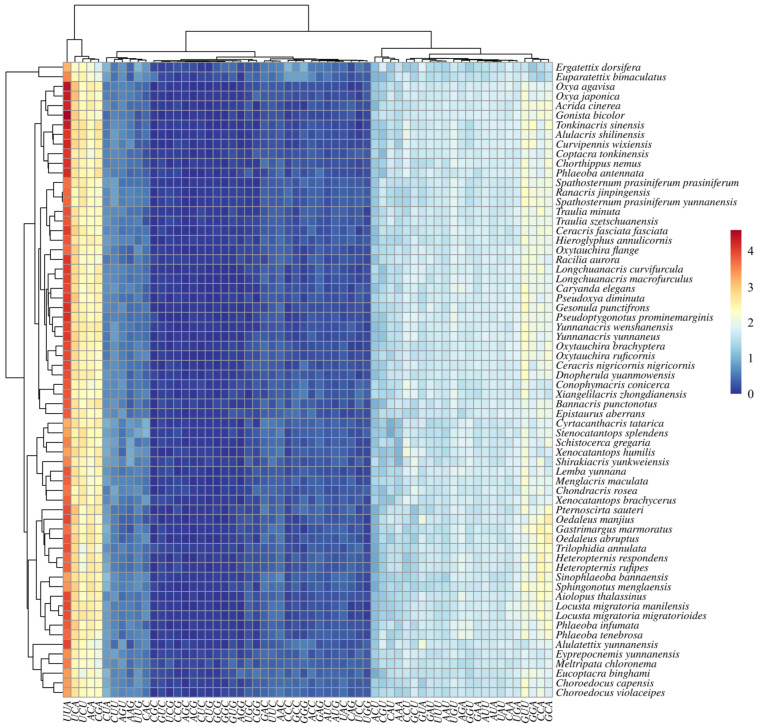
Hierarchical clustering analysis of relative synonymous codon usage (RSCU) values in mitochondrial protein-coding genes across Acrididae species. The heatmap shows RSCU values for all codons (x-axis) in each species (y-axis). Colors represent RSCU values ranging from 0 (dark blue) to 4 (dark red), with white indicating neutral usage (RSCU = 1). The dendrogram on the left shows the clustering of species based on their codon usage patterns.

To provide additional evidence supporting our phylogenetic findings, we conducted comprehensive codon usage and genetic distance analyses across all these Acrididae mitochondrial genomes. The relative synonymous codon usage (RSCU) values were calculated for all protein-coding genes, revealing consistent patterns in codon preference across species (Figure 7). The hierarchical clustering analysis of RSCU values demonstrated that all examined Acrididae species, including our newly sequenced specimens, exhibited similar codon usage patterns. A strong preference for A/T-ending codons over G/C-ending codons was consistently observed across all species. Specifically, NNA and NNU codons showed significantly higher RSCU values (generally >1.0) compared to NNG and NNC codons (generally <1.0). For instance, the leucine codons UUA and CUA were used more frequently than UUG and CUG, while for arginine, AGA and CGA were preferred over AGG and CGG.

This consistent bias in codon usage appears to be a conserved feature across Acrididae mitochondrial genomes. However, our analysis showed that several groups exhibited distinct codon usage signatures. The position of *Conophymacris conicerca* (Conophyminae) in our analyses challenges its current subfamilial placement. This species consistently groups with members of the Oxyinae clade rather than with its traditionally assigned subfamily members, suggesting a potential misclassification. Similarly, the unexpected clustering of *Spathosternum prasiniferum* (Spathosterninae) with members of the Oxyinae indicates that the boundaries between these subfamilies may need reconsideration.

Moreover, our results also highlight potential paraphyly within the Coptacrinae. While *Eucoptacra binghami*, *Epistaurus aberrans*, and *Coptacra tonkinensis* form a well-supported group, their relationship with other presumed Coptacrinae members is not strongly supported. The *Pseudotraulia cornuata* displayed a unique codon usage pattern that differed from its previously assumed closest relatives, providing additional support for its taxonomic revision. Additionally, the newly sequenced *Longchuanacris macrofurculus* and *Lemba yunnana* form a distinct clade that shows closer affinity to the Oxyinae than to their currently assigned subfamilies, warranting careful taxonomic reconsideration.

**Figure 8:**
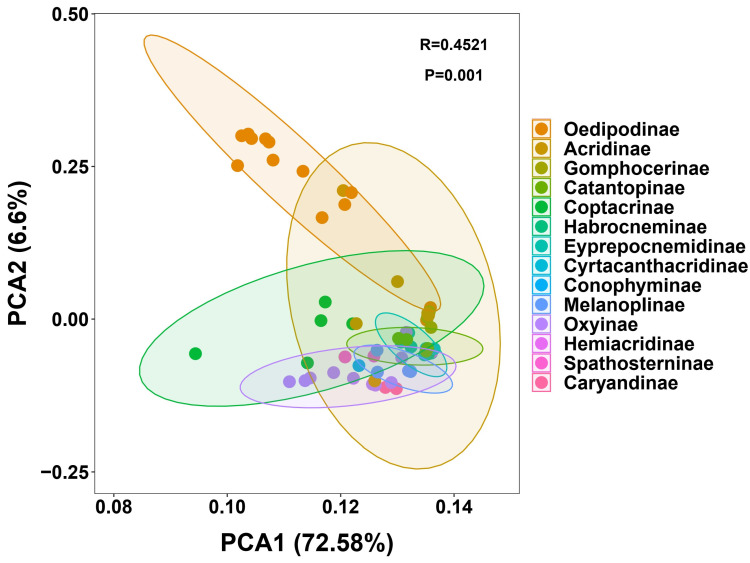
Principal component analysis (PCA) of genetic distances among 63 Acrididae species representing 13 subfamilies. The plot shows the first two principal components. Different colors represent distinct subfamilies, with ellipses indicating 95% confidence intervals for each subfamily group. Points represent individual species. The significant clustering pattern (R = 0.4521, *p* = 0.001) demonstrates clear subfamily-level genetic differentiation.

The genetic distances were calculated using the concatenated alignment of 13 mitochondrial protein-coding genes and 16S rRNA gene sequences, providing a comprehensive dataset for evolutionary distance estimation. Based on the retrieved matrix, we initially performed principal component analysis (PCA). The first two principal components explained a large proportion of the total variance, with PC1 and PC2 accounting for 72.58% and 6.6% of the variation, respectively (Figure 8). The PCA results revealed clear clustering patterns at the subfamily level (R = 0.4521, *p* = 0.001). Notably, the Oedipodinae formed a distinct cluster in the upper left quadrant of the plot, showing clear separation from other subfamilies. This distinct clustering suggests substantial genetic divergence among Oedipodinae and other subfamilies within Acrididae. The Catantopinae showed a relatively wide distribution in the PCA plot, with its 95% confidence ellipse overlapping with several other subfamilies, particularly the Cyrtacanthacridinae and Melanoplinae. This pattern suggests closer genetic relationships among these three subfamilies compared to others, which is consistent with their morphological similarities. Several smaller subfamilies, including Oxyinae, Hemiacridinae, and Spathosterninae, formed more compact clusters in the central region of the plot, indicating relatively conserved genetic distances within these groups. The Acridinae showed an intermediate distribution pattern, with some overlap with both the Oedipodinae and Catantopinae clusters.

**Figure 9:**
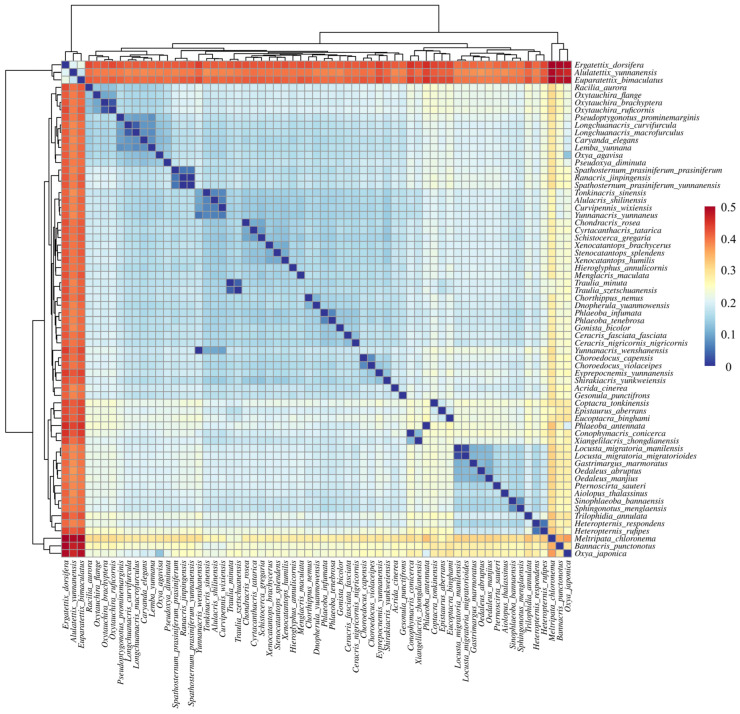
Pairwise genetic distances among 63 Yunnan Acrididae species based on concatenated mitochondrial sequences (13 protein-coding genes and 2 RNA genes). Hierarchical clustering heatmap showing genetic distances between species pairs. The color gradient from blue to red represents increasing genetic distance (scale bar shown).

As per our concerns to validate our phylogenetic findings, we further clustered these genetic distances. The analysis revealed considerable sequence divergence among the studied species, with interspecific distances ranging from 0.036 to 0.529 (mean = 0.193) (Figure 9). The analysis revealed several major clusters with varying degrees of genetic divergence. The most distinctive cluster comprises members of the Oedipodinae, including *Heteropternis rufipes*, *Trilophidia annulata*, and *Pternoscirta sauteri*, showing relatively low within-group genetic distances (0.1–0.2) but high between-group distances (0.3–0.4) with other clusters. This pattern supports the monophyly of this subfamily while highlighting its substantial evolutionary divergence from other grasshopper lineages.

A second major cluster includes representatives from the Catantopinae and related subfamilies, with *Xenocatantops humilis* and its allies showing intermediate levels of genetic differentiation (0.2–0.3). Notably, some species traditionally assigned to this group show unexpected patterns of genetic similarity with members of other subfamilies, suggesting potential taxonomic inconsistencies. The newly sequenced *Longchuanacris macrofurculus* and *Lemba yunnana* form a distinct subcluster with relatively low genetic distances between them (0.1–0.15), supporting their close evolutionary relationship.

The analysis also revealed several interesting patterns among the Oxyinae and Spathosterninae representatives. *Spathosternum prasiniferum* shows closer genetic affinity to members of the Oxyinae than to its traditionally assigned subfamily members, with genetic distances ranging from 0.15 to 0.25. This finding supports our phylogenetic results, suggesting potential misclassification of this taxon. Several species show unique patterns of genetic differentiation that do not clearly align with current taxonomic classifications. For example, *Conophymacris conicerca* displays intermediate genetic distances (0.25–0.35) to multiple groups, suggesting a complex evolutionary history. The analysis also highlights significant genetic divergence among geographically isolated populations of widespread species, indicating potential cryptic diversity within these taxa.

The genetic distance analysis provided additional support for some of our proposed taxonomic revisions. For instance, *Pseudoptygonotus prominemarginis* exhibited relatively high genetic distances (>0.20) from its previously assumed closest relatives, corroborating our phylogenetic results and suggesting its distinct evolutionary position. Similarly, the genetic distances observed for *Spathosternum prasiniferum* and *Xenocatantops humilis* aligned well with their proposed taxonomic placements based on morphological and phylogenetic analyses. On a broader scale, the hierarchical clustering of genetic distances revealed clear patterns of evolutionary divergence across the family Acrididae (Figure 9). These patterns generally corresponded well with traditional subfamily classifications while also highlighting potential areas where taxonomic revision might be warranted, particularly among some of the newly sequenced Yunnan specimens.

## 4. Discussion

In this presented study, the comprehensive analysis of mitochondrial genomes from 17 grasshopper species of the Acrididae family in Yunnan Province, combined with data from an additional 46 previously reported species, has yielded significant insights into the phylogenetic relationships, divergence times, and evolutionary dynamics within this diverse group. Our findings not only advance the systematic understanding of Acrididae in one of the world’s most biodiverse regions but also illuminate broader patterns of insect evolution and biodiversity maintenance.

### 4.1. Phylogenetic Relationships and Taxonomic Revisions

One of the most striking outcomes of our study is the revelation of several phylogenetic incongruences with traditional morphology-based classifications. Our phylogenetic framework provides a robust foundation for taxonomic revisions, advocating for the integration of molecular markers to refine and possibly redefine subfamilies and generic classifications within Acrididae. Notably, *Conophymacris conicerca* is currently classified within the Dericorythidae family rather than Acrididae, and our findings are consistent with this updated classification [37]. Similarly, species such as *Spathosternum prasiniferum* and *Longchuanacris macrofurculus*, along with *Lemba yunnana*, exhibit clustering patterns that challenge their current taxonomic placements within the Oxyinae and other subfamilies. These findings corroborate with the earlier molecular studies, which have similarly highlighted the polyphyletic and paraphyletic nature of these subfamilies within Acrididae [37]. However, we must cautiously state that all these findings of taxonomic revisions have mostly been brought up by previous research; we just confirmed them using additional molecular sequences from newly sequenced species.

The monophyly of subfamilies like Oedipodinae and Coptacrinae is strongly supported, aligning with their morphological coherence [38,39]. However, the observed placement of some Coptacrinae, such as *Pseudotraulia cornuata* representatives, suggests potential paraphyly, necessitating a reevaluation of subfamily boundaries. This underscores a broader trend in entomological taxonomy, where molecular data increasingly reveal the limitations of morphology-based classifications, particularly in groups with subtle morphological distinctions and high species diversity.

The hierarchical clustering of genetic distances further corroborates these insights, revealing distinct evolutionary clusters and highlighting potential cryptic diversity within certain taxa. For instance, *Pseudoptygonotus prominemarginis* exhibits high genetic distances from its presumed relatives, supporting its placement as a distinct lineage. Furthermore, the PCA of genetic distances underscores clear genetic differentiation at the subfamily level, validating the phylogenetic clades identified. The pronounced clustering of Oedipodinae species, in particular, reflects substantial genetic divergence from other subfamilies, aligning with their well-supported monophyly in phylogenetic trees. Conversely, the overlapping distributions of Catantopinae, Cyrtacanthacridinae, and Melanoplinae in the PCA plot suggest closer genetic relationships, possibly indicative of shared evolutionary histories [4,19].

In addition, based on detailed morphological examinations [40], our ongoing taxonomic revision of Yunnan grasshoppers strongly indicates that *Ranacris jinpingensis* should be synonymized with *Ranacris yunnanensis*. The comprehensive morphological analysis reveals no significant diagnostic characters that could reliably distinguish these two nominal species. To maintain nomenclatural stability and facilitate future research, we propose to treat *R. jinpingensis* as a junior synonym of *R. yunnanensis* in this study. This taxonomic decision is particularly important for the continued utility of the mitochondrial genome data presented here. The morphological similarities between these taxa, combined with their geographic distribution patterns and ecological preferences, further support this synonymization [40]. This taxonomic adjustment will be formally presented in our forthcoming systematic revision of Yunnan Acrididae fauna. Therefore, throughout this paper, we refer to this species as *R. yunnanensis* to ensure consistency with the future literature and to prevent potential confusion in subsequent molecular studies utilizing these mitochondrial genome sequences.

Although generally corresponding with previous phylogenetic trees [4,37], our phylogenetic analysis revealed a notably fragmented distribution of Acridinae species across different clades, challenging the traditional subfamily classification within Acrididae. Specifically, three Acridinae species in our study—*Sinophlaeoba bannaensis*, *Aiolopus thalassinus*, and *Pseudoptygonotus prominemarginis*—were recovered in distinctly separate clades, with *S. bannaensis* clustering within Oedipodinae, while *A. thalassinus* and *P. prominemarginis* were placed in other divergent positions. This widespread non-monophyly of Acridinae taxa suggests fundamental issues with the current subfamily classification. Several factors may explain these unexpected phylogenetic placements. First, the morphological characters traditionally used to define Acridinae (such as the presence of lateral carinae on the pronotum and specific features of the fastigium) may represent homoplastic traits that evolved independently multiple times rather than reliable synapomorphies. Second, the dispersed distribution of Acridinae members across different clades in our phylogeny strongly indicates that this subfamily, as currently defined, is polyphyletic and requires comprehensive taxonomic revision. This finding aligns with recent molecular studies that have increasingly questioned the monophyly of Acridinae [3].

### 4.2. Divergence Times and Evolutionary Dynamics

The molecular dating analysis situates the diversification of Yunnan Acrididae within a temporal framework that aligns with significant geological and climatic events. The crown age of approximately 51.94 million years ago (Mya) places the initial diversification in the Early Eocene, a period characterized by global warming and substantial tectonic activity, including the uplift of the Tibetan Plateau [41,42]. This timing suggests that climatic and geographical changes played pivotal roles in driving speciation and diversification within Acrididae.

Subsequent radiations during the Late Eocene and Middle Oligocene correspond to further uplift and climatic shifts, including the formation of the modern Asian monsoon system [41,43]. These events likely generated diverse habitats and environmental gradients, fostering both allopatric and ecological speciation. The intense diversification observed during the Miocene and the Pliocene–Pleistocene transitions highlights the influence of climatic oscillations and habitat diversification on recent speciation events [1,44,45]. These temporal patterns of diversification not only elucidate the evolutionary history of Acrididae in Yunnan but also contribute to our understanding of how historical climatic and geological processes shape current biodiversity patterns. The correlation between diversification events and environmental changes underscores the dynamic interplay between organisms and their habitats, a fundamental principle in evolutionary biology.

Coupled with our previous research, as documented in the book ‘Fauna and Distribution Patterns of Yunnan Grasshoppers’ [40], we have proposed two main mechanisms underlying the exceptional diversity of Acrididae in Yunnan Province: multiple origins and species diversification. The latter was hypothesized to have occurred during the past 5.5 million years, driven by geographical and ecological isolation resulting from the uplift of the Tibetan Plateau. Our molecular phylogenetic analysis not only validates these hypotheses but also provides precise temporal frameworks for lineage diversification. The divergence time estimation reveals that while the initial radiation of Yunnan Acrididae began in the Early Eocene (approximately 51.94 Mya), significant speciation events indeed occurred during the Pliocene (~5.63 Mya), aligning remarkably well with previous predictions. This recent diversification phase coincides with the intensified uplift of the Tibetan Plateau, which dramatically altered the regional topography and climate patterns. Our findings demonstrate that this geological event triggered both allopatric speciation through geographical barriers and ecological speciation via the creation of novel habitat types and climatic zones. The fine-scale temporal resolution provided by our molecular data further reveals that different Acrididae lineages responded asynchronously to these environmental changes, with some groups (e.g., Coptacrinae) showing accelerated diversification rates during this period. This temporal framework not only confirms previous hypotheses about the timing of grasshopper diversification in Yunnan but also provides unprecedented detail about the sequence and tempo of speciation events across different taxonomic groups.

### 4.3. Species Conservation Implications

Our phylogenetic analyses reveal several evolutionarily distinct lineages that warrant immediate conservation attention. Among the newly documented species in this study, several taxa show extremely restricted distributions, being rarely observed even in neighboring provinces such as Guangxi. These include species such as *Ranacris jinpingensis* and *Eucoptacra binghami* that are notably difficult to observe and collect [37]. While we have maintained a conservative approach by not explicitly declaring these as Yunnan endemics due to the possibility of future discoveries in adjacent regions, their current known distributions suggest a high conservation priority.

Of particular concern are the montane-distributed species confirmed as Yunnan endemics [46]. The *Longchuanacris-Lemba* clade exhibits significant genetic divergence and highly restricted distributions, making these taxa especially vulnerable to habitat loss and environmental change. Their endemic status, combined with their genetic distinctiveness, underscores their importance for conservation planning [47,48]. Our analyses revealed three habitat types of particular conservation significance in Yunnan. Mid-elevation montane regions above 1500 m exhibited exceptional endemism, with several species restricted to these elevational bands. The biogeographic transition zone between the eastern Himalayas and Yunnan plateau emerged as another critical area characterized by unique assemblages of grasshopper species [48,49]. Additionally, the ecotones between valleys and mountain slopes supported populations with distinct genetic signatures, likely resulting from long-term isolation. These findings suggest that future conservation efforts in Yunnan should focus on preserving these key habitats to maintain grasshopper diversity.

Based on our molecular findings, we propose three primary conservation strategies, which is also documented in our book ‘Fauna and Distribution Patterns of Yunnan Grasshoppers’ [40]. First, existing protected areas should be expanded to encompass identified diversity hotspots, particularly in mid-elevation zones where endemic species concentrate. Second, habitat connectivity should be maintained among populations of range-restricted species through carefully planned ecological corridors. Third, long-term genetic monitoring programs should be established for evolutionarily distinct lineages to track population health and genetic diversity. The integration of molecular data into conservation planning provides an objective framework for prioritizing protection efforts. Our results suggest that conservation strategies in Yunnan should focus on preserving both evolutionary distinctiveness and genetic diversity rather than simply maximizing species numbers. This approach would better preserve the evolutionary potential and ecological resilience of grasshopper communities in the region.

### 4.4. Mitochondrial Genome Conservation and Variation

Our analysis reveals a striking conservation of mitochondrial genome organization across the sampled Acrididae species, with no evidence of tRNA duplications or rearrangements. Only the total length slightly varies, from 15,403 to 15,943 bp. This high degree of synteny is consistent with patterns observed in other Acrididae species, where mitochondrial genome structure tends to be highly conserved despite significant morphological and ecological diversification [50,51]. Such conservation suggests strong evolutionary constraints on mitochondrial genome organization, possibly due to the essential roles these genomes play in cellular energy metabolism and other vital processes.

However, variations observed in specific genomic regions, such as longer CDS and rRNA genes in newly sequenced species, alongside higher GC content, indicate localized evolutionary pressures or adaptations [52,53]. These genomic variations might reflect responses to different environmental conditions or metabolic demands across Yunnan’s diverse ecological niches. The higher GC content, albeit subtle, could influence gene expression and mitochondrial function, potentially conferring adaptive advantages under varying thermal or metabolic stresses.

The codon usage patterns for all protein-coding sequences were also found with high conservation. The consistent preference for A/T-ending codons across Acrididae mitochondrial genomes highlights a conserved feature of codon usage bias in these insects. This bias may be driven by mutational pressures, genome-wide A/T richness, or selection for translational efficiency and accuracy [52,54]. However, the distinct codon usage signatures observed in certain species, such as *Conophymacris conicerca*, further support the phylogenetic anomalies revealed by our gene-based analyses, reinforcing the need for taxonomic reconsideration.

### 4.5. Implications for Future Studies

Our study leverages comprehensive mitochondrial genomic data to unravel complex phylogenetic relationships, underscoring the utility of mitochondrial DNA in systematic biology. The integration of mitochondrial genomes with advanced phylogenetic and statistical methods, such as ASTRAL and BEAST2, demonstrates a robust approach to resolving intricate evolutionary histories [55,56,57]. However, reliance solely on mitochondrial genomes can present limitations, including the potential for mitochondrial introgression, incomplete lineage sorting, and the maternal inheritance bias that might not accurately reflect nuclear genome diversity [58,59]. Future studies should complement mitochondrial data with nuclear genomic information to provide a more holistic view of Acrididae phylogeny. Techniques such as transcriptome sequencing or genome-wide single nucleotide polymorphism (SNP) analyses could offer deeper insights into gene flow, hybridization events, and the genomic basis of adaptive traits. As expertise in classical taxonomy becomes increasingly scarce, these advanced molecular methods provide a vital alternative for species identification and classification. Building comprehensive databases of mitochondrial and nuclear genomes, coupled with open-access platforms for data sharing and analysis, would facilitate large-scale comparative studies and accelerate discoveries in systematics and evolution [51,58,59,60].

## Figures and Tables

**Table 1:** The source of sample information.

Subfamily	Species	Authority	Collecting Data
Acrididae			
Acridinae	*Pseudoptygonotus prominemarginis*	Zheng and Mao, 1996	China: Yunnan: Dali (from Orthoptera Species File)
Caryandinae	*Lemba yunnana*	Ma and Zheng, 1994	China: Yunnan
Catantopinae	*Ranacris jinpingensis*	Zheng, Lin, Deng and Shi, 2015	China: Yunnan: Jinping
Catantopinae	*Xenocatantops humilis*	(Serville, 1838)	Baie de Palabaun
Conophyminae	*Conophymacris conicerca*	Bi and Xia, 1984	China: Yunnan: Baoshan
Coptacrinae	*Pseudotraulia cornuata*	Laosinchai and Jago, 1980	Thailand
Coptacrinae	*Coptacra tonkinensis*	Willemse, 1939	Vietnam: L?ng Son: Than-Moi
Coptacrinae	*Epistaurus aberrans*	Brunner von Wattenwyl, 1893	Myanmar: Kachin: Bhamó
Coptacrinae	*Eucoptacra binghami*	Uvarov, 1921	Myanmar
Coptacrinae	*Meltripata chloronema*	Zheng, 1982	China: Yunnan: Jinghong
Eyprepocnemidinae	*Choroedocus violaceipes*	Miller, 1934	Negri Sembilan, Tampin
Eyprepocnemidinae	*Eyprepocnemis yunnanensis*	Zheng, Lian and Xi, 1982	China: Yunnan: Jinghong
Oedipodinae	*Heteropternis rufipes*	(Shiraki, 1910)	Japan
Oedipodinae	*Pternoscirta sauteri*	(Karny, 1915)	Taiwan: Taiwan: Nantou: Kosempo
Oxyinae	*Longchuanacris macrofurculus*	Zheng and Fu, 1989	China: Yunnan: Ruili
Oxyinae	*Racilia aurora*	(Brunner von Wattenwyl, 1893)	Myanmar
Spathosterninae	*Spathosternum prasiniferum yunnanensis*	Wei and Zheng, 2005	China: Yunnan: Jinping, Mengla

## Data Availability

The assembled mitochondrial genomes were uploaded to the NCBI Genbank database under the IDs from OQ270615 to OQ270627. The detailed list can be found in Table S1.

## Supplementary Materials

The following supporting information can be downloaded at https://www.mdpi.com/article/10.3390/insects16020151/s1, Table S1: The Genbank accession of all mitogenomes used in this presented study.
