# Correction: Zhang et al. Phylogenetics and Evolutionary Dynamics of Yunnan Acrididae Grasshoppers Inferred from 17 New Mitochondrial Genomes. *Insects* 2025, *16*, 151

**DOI:** 10.3390/insects16090947

**Published:** 2025-09-10

**Authors:** Keyao Zhang, Jing Song, Junhui Lu, Lu Zhao, Weian Deng, Delong Guan, Benyong Mao

**Affiliations:** 1 College of Life Sciences, Shaanxi Normal University, Xi’an 710119, China; keyaozh@163.com (K.Z.); 2023660007@hcnu.edu.cn (J.S.); lujunhui@snnu.edu.cn (J.L.); zl609677869@snnu.edu.cn (L.Z.); 2 Guangxi Key Laboratory of Sericulture Ecology and Applied Intelligent Technology, Hechi University, Hechi 546300, China; dengweian5899@163.com; 3 Guangxi Collaborative Innovation Center of Modern Sericulture and Silk, Hechi University, Hechi 546300, China; 4 College of Agriculture and Biological Science, Dali University, Dali 671003, China

**Error in Figures 4–9**

In the original publication [1], there was a mistake regarding *Ranacris jinpingensis* in Figures 4–9 as published. A specimen was misidentified as *Ranacris jinpingensis* when it should have been identified as *Spathosternum prasiniferum yunnanensis*, leading to inaccuracies in the phylogenetic analysis. The corrected Figure 4, Figure 5, Figure 6, Figure 7, Figure 8 and Figure 9 with *Spathosternum prasiniferum yunnanensis1* appear below. The authors state that the scientific conclusions are unaffected. This correction was approved by the Academic Editor. The original publication has also been updated. 

**Figure 4:**
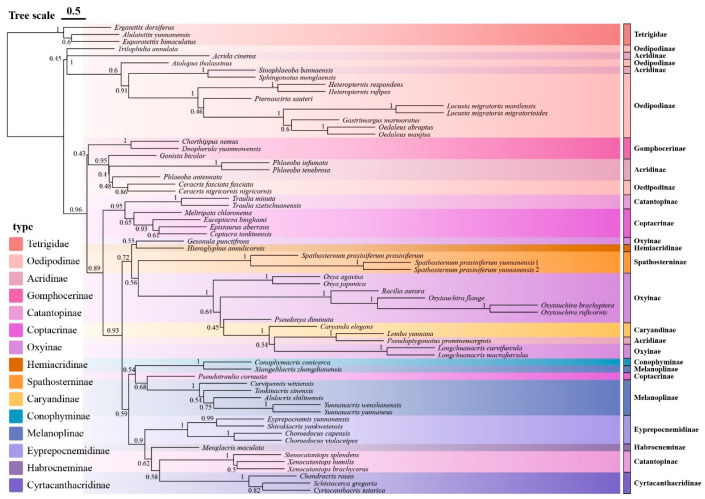
Species tree of Yunnan Acrididae based on concatenated mitochondrial genes. The tree was constructed using ASTRAL from individual gene trees generated by IQ-TREE maximum likelihood analyses. Numbers at nodes represent posterior probability values.

**Figure 5:**
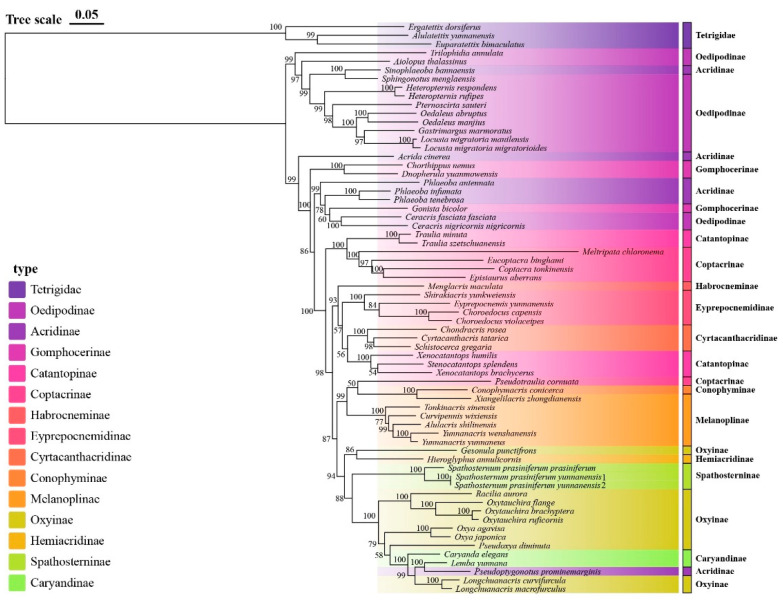
Maximum likelihood phylogenetic tree of Yunnan Acrididae based on concatenated mitochondrial sequences. Branch support values are shown as ultrafast bootstrap percentages (UFboot). Major subfamilies are indicated by different colors.

**Figure 6:**
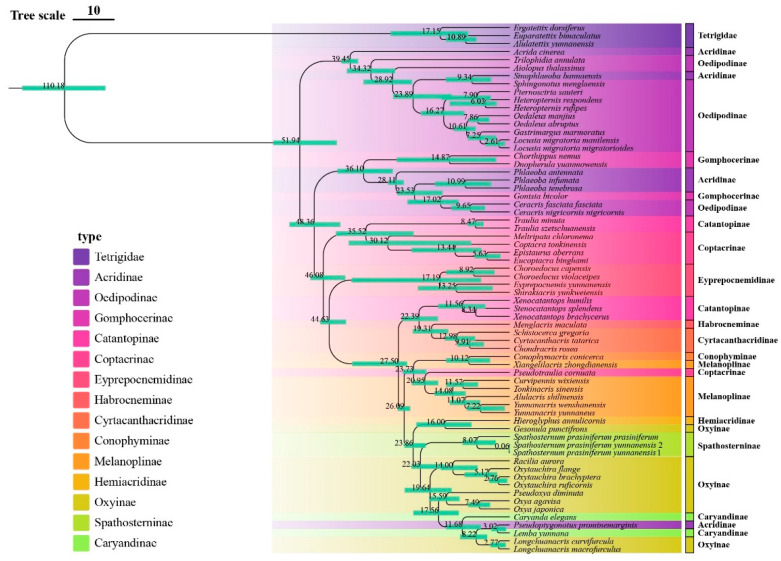
Time-calibrated phylogeny of Yunnan Acrididae based on concatenated mitochondrial sequences (13 PCGs + 2 rRNAs) using BEAST2. Node ages are shown in millions of years (Mya), with green bars indicating 95% highest posterior density (HPD) intervals.

**Figure 7:**
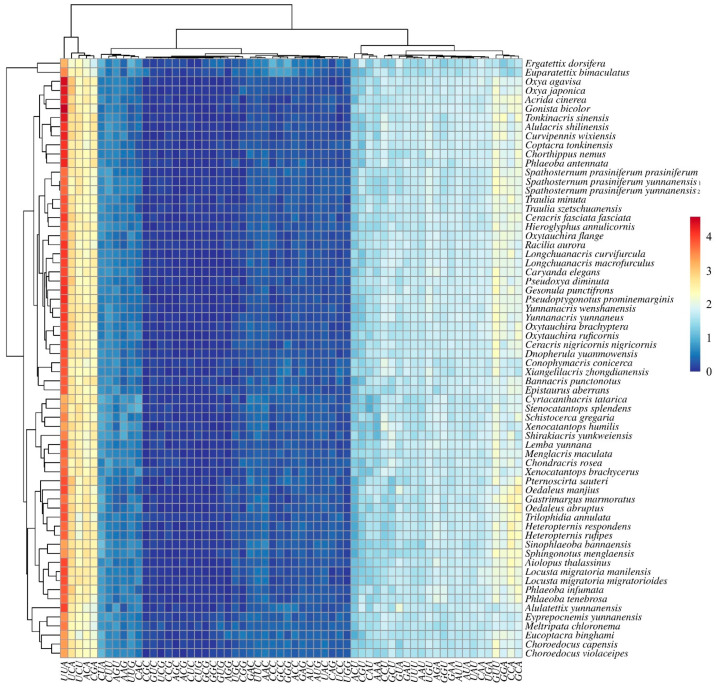
Hierarchical clustering analysis of relative synonymous codon usage (RSCU) values in mitochondrial protein-coding genes across Acrididae species. The heatmap shows RSCU values for all codons (x-axis) in each species (y-axis). Colors represent RSCU values ranging from 0 (dark blue) to 4 (dark red), with white indicating neutral usage (RSCU = 1). The dendrogram on the left shows the clustering of species based on their codon usage patterns.

**Figure 8:**
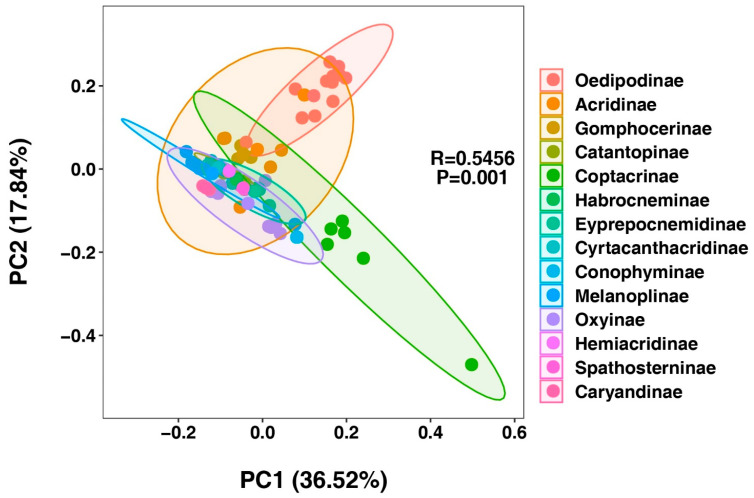
Principal component analysis (PCA) of genetic distances among 63 Acrididae species representing 13 subfamilies. The plot shows the first two principal components. Different colors represent distinct subfamilies, with ellipses indicating 95% confidence intervals for each subfamily group. Points represent individual species. The significant clustering pattern (R = 0.5456, *p* = 0.001) demonstrates clear subfamily-level genetic differentiation.

**Figure 9:**
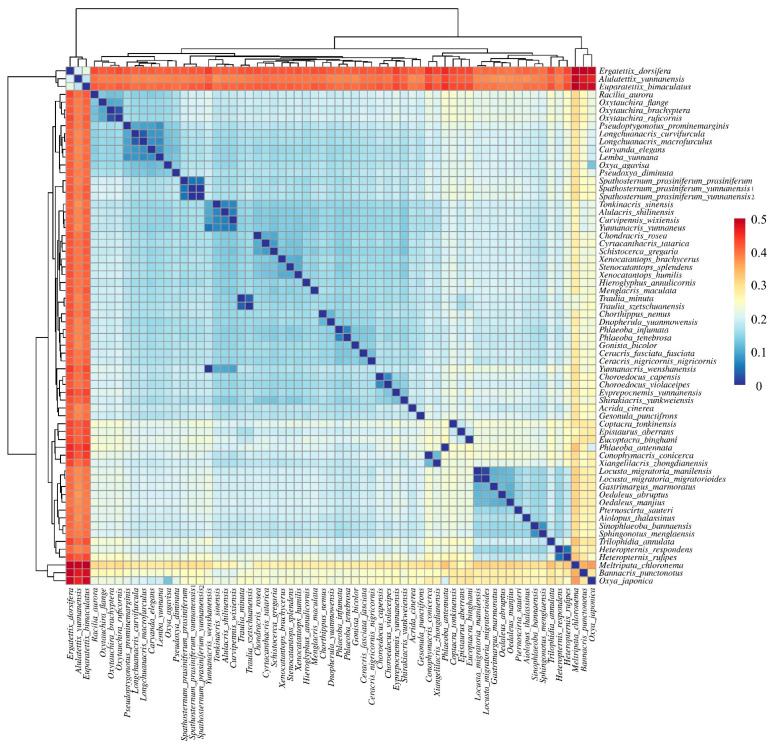
Pairwise genetic distances among 63 Yunnan Acrididae species based on concatenated mitochondrial sequences (13 protein-coding genes and 2 RNA genes). Hierarchical clustering heatmap showing genetic distances between species pairs. The color gradient from blue to red represents increasing genetic distance (scale bar shown).

**Error in Table 1**

In the original publication [1], there was a mistake in *Ranacris jinpingensis* of Table 1 as published. A specimen was misidentified as *Ranacris jinpingensis* when it should have been identified as *Spathosternum prasiniferum yunnanensis*, leading to inaccuracies in the phylogenetic analysis. The corrected Table 1 with *Spathosternum prasiniferum yunnanensis1* appears below. The authors state that the scientific conclusions are unaffected. This correction was approved by the Academic Editor. The original publication has also been updated.

**Table 1:** The source of sample information.

Subfamily	Species	Authority	Collecting Data
Acrididae			
Acridinae	*Pseudoptygonotus prominemarginis*	Zheng and Mao, 1996	China: Yunnan: Dali (from Orthoptera Species File)
Caryandinae	*Lemba yunnana*	Ma and Zheng, 1994	China: Yunnan
Spathosterninae	*Spathosternum prasiniferum yunnanensis1*	Wei and Zheng, 2005	China: Yunnan: Jinping
Catantopinae	*Xenocatantops humilis*	(Serville, 1838)	Baie de Palabaun
Conophyminae	*Conophymacris conicerca*	Bi and Xia, 1984	China: Yunnan: Baoshan
Coptacrinae	*Pseudotraulia cornuata*	Laosinchai and Jago, 1980	Thailand
Coptacrinae	*Coptacra tonkinensis*	Willemse, 1939	Vietnam: Son La: Than-Moi
Coptacrinae	*Epistaurus aberrans*	Brunner von Wattenwyl, 1893	Myanmar: Kachin: Bhamó
Coptacrinae	*Eucoptacra binghami*	Uvarov, 1921	Myanmar
Coptacrinae	*Meltripata chloronema*	Zheng, 1982	China: Yunnan: Jinghong
Eyprepocnemidinae	*Choroedocus violaceipes*	Miller, 1934	Negri Sembilan, Tampin
Eyprepocnemidinae	*Eyprepocnemis yunnanensis*	Zheng, Lian and Xi, 1982	China: Yunnan: Jinghong
Oedipodinae	*Heteropternis rufipes*	(Shiraki, 1910)	Japan
Oedipodinae	*Pternoscirta sauteri*	(Karny, 1915)	Taiwan: Taiwan: Nantou: Kosempo
Oxyinae	*Longchuanacris macrofurculus*	Zheng and Fu, 1989	China: Yunnan: Ruili
Oxyinae	*Racilia aurora*	(Brunner von Wattenwyl, 1893)	Myanmar
Spathosterninae	*Spathosternum prasiniferum yunnanensis2*	Wei and Zheng, 2005	China: Yunnan: Jinping, Mengla

**Text Correction**

There was an error in the original publication [1]. A specimen of *Spathosternum prasiniferum yunnanensis* was misidentified as *Ranacris jinpingensis*. We have updated all occurrences of this species name throughout the text, including (1) Paragraph 1 of Section 2.1; (2) Paragraph 5 of Section 3.2; (3) Paragraph 4 of Section 4.1; and (4) Paragraph 1 of Section 4.3.

A correction has been made to 2. Materials and Methods, 2.1. Sample Collection and DNA Extraction.

Corrected paragraph: Specimens representing 17 Acrididae species were collected from various locations across Yunnan Province, China, between 2018 and 2023 by Prof. Maobenyong’s team at Dali University (Table 1). Collection sites covered diverse ecological zones across the entire Yunnan Province, which does not have major biogeographic zones. However, these species showed strong zone specificity—for example, *Eyprepocnemis yunnanensis* was found exclusively in the southern region of Yunnan. Collection sites for these species were strategically selected to represent all three major biogeographic zones within Yunnan: The northwestern localities of Dali and Baoshan, the southeastern localities of Jinping and Mengla, and the southern valleys of Jinghong and Ruili. For each species, over three individuals were collected. All specimens were morphologically identified using established taxonomic keys and preserved in 100% ethanol at −80 °C. For DNA sequencing preparation, one sample with the largest body size was selected from each species collection. Total genomic DNA was extracted from the muscle tissue of the femur using the TIANamp Genomic DNA Kit (TIANGEN, Beijing, China. DP304-03) following the manufacturer’s protocols.

A correction has been made to 3. Results, 3.2. Phylogenetic Inference and Divergence Time Estimation

Corrected paragraph: The phylogenetic reconstruction demonstrates that it has uplifted most of the hierarchical clades with strong bootstrap support (>90%). The Oedipodinae clade, including *Locusta*, *Gastrimargus*, *Oedaleus*, and related genera, is strongly supported (97–100%). Within this group, the two subspecies of *Locusta migratoria* (*L. m. manilensis* and *L. m. migratorioides*) show an extremely close relationship with very short branch lengths. Another well-supported clade (100%) comprises the *Oxya* species (*O. agavisa* and *O. japonica*), sister to *Pseudoxya diminuta*. The *Oxytauchira* species form a monophyletic group with 100% bootstrap support, with *O. brachyptera* and *O. ruficornis* being more closely related to each other than to *O. flange*. The genus *Yunnanacris* (*Y. wenshanensis* and *Y. yunnaneus*) forms a well-supported clade (100%), sister to *Alulacris shilinensis* (99%).

A correction has been made to 4. Discussion, 4.1. Phylogenetic Relationships and Taxonomic Revisions.

Corrected paragraph: In addition, based on detailed morphological examinations [40], our ongoing taxonomic revision of Yunnan grasshoppers provides new insights into their phylogeny.

A correction has been made to 4. Discussion, 4.3. Species Conservation Implications

Corrected paragraph: Our phylogenetic analyses reveal several evolutionarily distinct lineages that warrant immediate conservation attention. Among the newly documented species in this study, several taxa show extremely restricted distributions, being rarely observed even in neighboring provinces such as Guangxi. These include species such as *Eucoptacra binghami* that are notably difficult to observe and collect [37]. While we have maintained a conservative approach by not explicitly declaring these as Yunnan-endemic species due to the possibility of future discoveries in adjacent regions, their current known distributions suggest a high conservation priority.

The authors state that the scientific conclusions are unaffected. This correction was approved by the Academic Editor. The original publication has also been updated.
